# Investigations Concerning Heavy Metals Dynamics in *Reynoutria japonica* Houtt.-Soil Interactions

**DOI:** 10.3390/toxics11040323

**Published:** 2023-03-29

**Authors:** Roxana Vidican, Tania Mihăiescu, Anca Pleșa, Anamaria Mălinaș, Bianca-Alexandra Pop

**Affiliations:** 1 Department of Plant Culture, Faculty of Agriculture, University of Agricultural Sciences and Veterinary Medicine, 400372 Cluj-Napoca, Romania; 2 Department of Environmental Engineering and Protection, Faculty of Agriculture, University of Agricultural Sciences and Veterinary Medicine, 400372 Cluj-Napoca, Romania

**Keywords:** *Reynoutria japonica* Houtt., translocator factor, bioaccumulation factor, phytostabilizer, phytoextractor

## Abstract

*Reynoutria japonica* Houtt (RJ) is an extremely invasive plant species, found nowadays in a wide range of habitats, including those polluted with heavy metals (HM). The aim of this study was to investigate HM dynamics in RJ–soil interactions in five habitats historically polluted with HM located in Baia Mare city, Romania. The concentrations of major metal elements (Cd, Cu, Pb, Zn) in plant tissues (roots, stems, leaves) and soil samples collected from the study sites were analyzed via portable ED-XRF spectroscopy (converted), and the translocation factor (TF) and bioconcentration factor (BCF) were calculated. The mean values of HM in soil samples collected from the study sites exceeded the threshold limit values established by Romanian legislation. Generally, the highest concentration of Cd was recorded in the above-ground part of the plant (stem and leaves), while for Cu, Pb and Zn, the highest values (with few exceptions) were recorded in the root. The metal transfer was highly effective from soil to RJ, such that all four of the HM studied exceeded the normal range of metals in a plant. Analysis of metal concentrations in plant tissues showed an efficient movement of Cd and Zn to the above-ground parts of the plant, a tendency particularly pronounced in the case of Cd (TF and BCF > 1), while Pb was the least bioaccumulated HM. It may be concluded that RJ is able to tolerate high concentrations of HM, being a good phytoextractor for Cd and Zn.

## 1. Introduction

*Reynoutria japonica* Houtt. (RJ), also known as the Japanese Knotweed, is a herbaceous perennial geophyte native to eastern Asia, largely widespread in North America and Europe, including Romania. The expansion of this extremely invasive weed is of particular concern because of its ability to rapidly colonize diverse environments, especially anthropized habitats [1]. RJ is included in the top 100 world’s worst invasive alien species [2], and it is almost impossible to eradicate once established in a certain environment. In Romania, its presence was almost undocumented until 70 years ago, while at present, it is found in most of the riparian ecosystems from Transylvania, Maramureș, Crișana and Moldova, being an important threat to native biodiversity. Thanks to its versatility, nowadays, RJ is found in a wide range of habitats, including those polluted with HM, where only a few plant species can survive (including brownfields). 

Soil pollution by HM has become a serious problem in the world [3] because it affects living organisms at the whole ecosystem level [1] due to the biologically available fractions of metals that can pose toxicity risks to living organisms [4]. According to the European Environment Agency (EEA), approximately 2.5 million sites located in Europe are potentially contaminated by HM and organic contaminants and are likely to require remediation [5]. Heavy metal contamination has captured community attention, leading to increasing public awareness and research regarding metal toxicity and persistence in both natural ecosystems and brownfields [6]. This hazardous event may serve as a direct and negative filter on both the soil and plant communities, resulting in a community that is resilient to metals and likely capable of mitigation, lowering effective metal concentrations in the soil [7]. 

The most promising and cost-effective solution for in situ remediation of HM-contaminated soil [8,9,10] is the phytoremediation technique, which would provide an eco-friendly approach through the use of plants to remove contamination from the soil, sediments, and water [11,12]. This remediation method relies on some plant species’ native ability to deal with HM stress as a strategy for survival [13]. Two main adaptive strategies were defined by Baker [14], namely either by exclusion (the toxic effect is restricted to the roots, where it is detoxified, while the aerial parts remain more or less unaffected [15]) or by accumulation by indicators or hyperaccumulators (the HMs are translocated in their above-ground parts without phytotoxicity symptoms). The entire spectra of processes involved in the phytoremediation of HM from the contaminated sites include the translocation, accumulation, transport, transformation and volatilization of HM [16,17]. The best plant species candidates for phytoremediation should have a good tolerance to high concentrations of HM, an extended root system for exploring large soil volumes, a rapid growth rate, and high biomass potential [18,19].

Nowadays, heavy metal pollution affects a wide range of soils, including urban and agricultural ones. The major causes for the enrichment of HM in urban soils have been attributed to human activities such as vehicle traffic, industry, and mining activities [20]. One such example of a heavy metal-contaminated site is located in Baia Mare, one of the most populous cities in northern Romania. HM soil pollution (mainly by Cu, Cr, Pb, and Zn) found in Baia Mare resulted from the mining and metallurgical industries, and it is well recognized today, posing a significant risk to human health and the environment. 

An interesting fact related to the sites polluted with HM from Baia Mare is that among the very few plant species which managed to cope with the high HM concentration found in soils, RJ is included. Moreover, it seems that none of the methods tried so far to eradicate it have worked, as this species continues to proliferate in large areas from Baia Mare. Therefore, based on RJ’s ability to “rise from the ashes”, we could hypothesize that this plant species could have a certain remediation capacity. A limited number of studies have reported aspects concerning RJ′s ability to accumulate high amounts of HM so far, but no clear correlations between HM concentrations in soil and plant tissue have been established yet [1,21]. Moreover, to the best of our knowledge, there is a lack of sufficient information about RJ behavior in brownfields. Following this hypothesis, the aim of our research is to investigate HM dynamics in RJ-soil interactions, having as the main objectives to: (i) Investigate the concentrations of major metal elements (Cu, Cd, Pb, Zn) in soil samples collected from five study sites historically polluted with HM; (ii) Investigate the concentrations of major metal elements (Cu, Cd, Pb, Zn,) in RJ plant tissues (roots, stems, leaves) collected from the same five study sites located in Baia Mare city; (iii) Evaluate the potential of RJ to accumulate HM from soils and to what extent; (iv) Examine the HM dynamics in RJ-soil interactions through translocation factor (TF) and bioconcentration factor (BCF) assessment.

## 2. Materials and Methods

### 2.1. Sites Description

**Figure 1:**
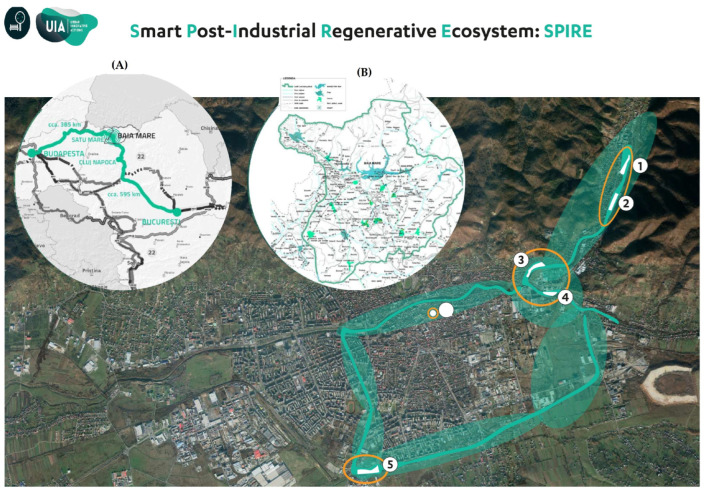
Location of the study sites. (**A**) The geographical location of Baia Mare city within Romania. (**B**) The Metropolitan area Baia Mare. (1) Romplumb. (2) Ferneziu. (3) Colonia Topitorilor. (4) Urbis. (5) Craica. (Source: Reproduced with permission from UIA SPIRE Baia Mare. Graphics Urbasofia).

The experiments were conducted on five sites located inside the town of Baia Mare (47°39′ N 23°34′ E) in North-West Romania, covering a total area of 7.3 hectares of brownfields (Figure 1). 

**Figure 2:**
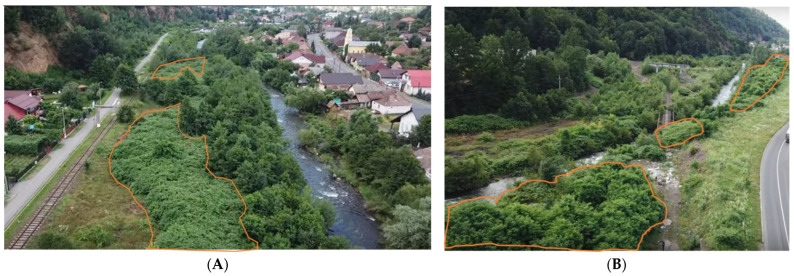
*Reynoutria japonica* Houtt. occurrence in Ferneziu (**A**), and Romplumb (**B**) sites. The areas marked with an orange line represent the area covered by RJ. (Source: Original).

The sites are characterized by different levels of soil HM contamination as a result of anthropogenic pollution, mainly by mining, the metallurgical industry, and urbanization. These sites were selected based on their soils’ high concentration of HM and the widespread presence of RJ species (Table 1; Figure 2). 

The major sources of HM pollution in Baia Mare city consist of two former metallurgical companies, namely Romplumb Baia Mare (a former state-owned company from Romania having Pb production as its main activity) and Cuprom Baia Mare (one of the highest producers of electrolytically refined copper (99.99% Cu) from Eastern Europe). Thanks to their location in the neighborhood of these two companies, the sites are recognized as highly polluted, especially with Cd, Cu, Pb and Zn. 

The sites consist of five riparian ecosystems characterized as brownfields (Table 1). The soil type is an anthropic protosoil (anthropic/urban soil), extensively influenced by human activities and previously used for industrial purposes. The vegetation found in these sites is characteristic of the riparian habitats since these sites are located close to Firiza River, mainly consisting of a few exemplars of *Salix* spp. and *Robinia pseudocacia* species and some grassland species like *Agrostis capillaris*. Large areas of these sites are covered by RJ, with the highest spread in Urbis and Ferneziu (Table 1). 

### 2.2. Soil Sampling and Analysis

Soil samples for chemical analyses were taken from the five experimental sites considered in this study. Soil samples were collected from the organic accumulation layer (A; top 10 cm) that represents the main rooting area of most plant species, including RJ, and from different areas among one experimental site (5 samples/each site, forming a composite sample), such that we can achieve accurate results. At the laboratory, the samples were cleaned to remove any debris (such as leaves, twigs, grass, and stones) and then air-dried by spreading them out on paper and exposing them to room temperature. Further, the soil samples were ground and sieved to reduce the particle size to less than 0.125 mm, homogenized and then analyzed for their HM concentration (Cd, Cu, Pb and Zn) using a Portable X-ray Fluorescence (XRF) spectroscope. Soil chemical analysis was also performed to determine the significant characteristics of the soils, such as pH, humus content, total nitrogen (N), and mobile phosphorous (P; performed and delivered by The Office for Pedological and Agrochemical Studies Cluj-Napoca). The determination of soil physico-chemical properties was performed according to the Romanian standards for soil quality, as follows: The pH—according to SR ISO 10390:2021 [22]; Organic carbon (organic C)—according to the method of wet oxidation (after Walkley–Black modified by Gogoașă, 1959 [23]; STAS 7184/21-82); Total nitrogen (N) after the Kjeldahl method [24]; Mobile phosphorus—after the Egner–Riehm-Domingo method [25].

### 2.3. Plant Sampling and Analysis

Plant samples consisting of RJ plant tissues (roots, stems, and leaves) were collected from all of the experimental sites studied in this research. Samples were collected from different areas among one experimental site (5 samples/each site forming a composite sample) to reach accurate results. Furthermore, plant samples were air-dried (following the same procedure described for soil samples) and subsequently ground with a special laboratory mill—dedicated to plant preparation. Furthermore, the samples were homogenized and then analyzed for HM concentration (for Cd, Cu, Pb and Zn) using a portable XRF.

Both soil and plant samples were collected twice per year, in spring 2021 (June) and autumn 2021 (October), and the results presented in this paper represent the average of these two sampling periods. Soil and RJ samples were collected from different distances from the two industrial sites (which represent the primary source of contamination), as follows: 10–100 m from Romplumb Baia Mare company for samples collected from Romplumb and Ferneziu experimental sites and 500–5000 m from the Cuprom Baia Mare company (the second major source of contamination), for samples collected from Colonia Topitorilor, Urbis and Craica experimental sites.

Before this research, a previous study was conducted to convert the data recorded by XRF to accurate quantitative data through linear regression, using the correlation between XRF and atomic absorption spectroscopy (AAS) data (data not published). This approach was successfully demonstrated by previous studies [26,27] as being effective for delivering accurate XRF data. 

### 2.4. Calculations and Data Statistical Analysis

#### 2.4.1. HM Dynamics in *Reynoutria japonica* Houtt.–Soil Interactions

The translocation (TF) and bioconcentration (BCF) indicators were calculated based on HM concentrations found in plant and soil samples. 

TF, also called the shoot-root quotient, describes the ability of a plant to translocate the metal from the roots through to the shoots and leaves and is generally used to validate if a plant species could be used as a candidate for phytoextraction [28].

The TF was calculated after the following formula [29]:

(1) $$TF=\frac{Cs}{Cr},$$

where: TF—translocation factor; Cs—metal concentration in shoots; Cr—metal concentration in the root.

The BCF reflects the potential of plants to regulate the uptake and mobility of HM in their tissues leading to its bioaccumulation in aerial parts [30].

The BCF was calculated after the following formula [30]:

(2) $$BCF=\frac{Cplant}{Csoil},$$

where: BCF—bioconcentration factor; Cplant—metal concentration in root, stem and leaf; Csoil—metal concentration in soil.

#### 2.4.2. Statistical Analysis

The effects of HM content on soil and plant tissues were assessed using descriptive statistics by Statistica vs. 10 (developed by StatSoft in 2010), t/F-test for single means and partial correlations and post hoc Tukey HSD from R statistical package [31]. Effects were accepted as statistically significant if *p* ≤ 0.05.

## 3. Results

### 3.1. Soil Chemical Composition and Content in Heavy Metal

Soil chemical composition revealed a wide range of soils among the five experimental sites studied in this research (Table 2). The pH ranged between very strong acid (Craica), moderate acid (Romplumb and Colonia Topitorilor) and neutral (Urbis). Variations are also observed for the Organic C, such that a very high content was recorded in Ferneziu, followed by Urbis. Very low organic C was found in Romplumb and Colonia Topitorilor. Concerning the content of essential plant nutrients, the chemical composition of soil revealed a good supply of N and P for Ferneziu (*p* < 0.001), while the values for the other sites varied between very poor nutrient content and an excessive supply of P in Urbis (*p* < 0.001).

**Figure 3:**
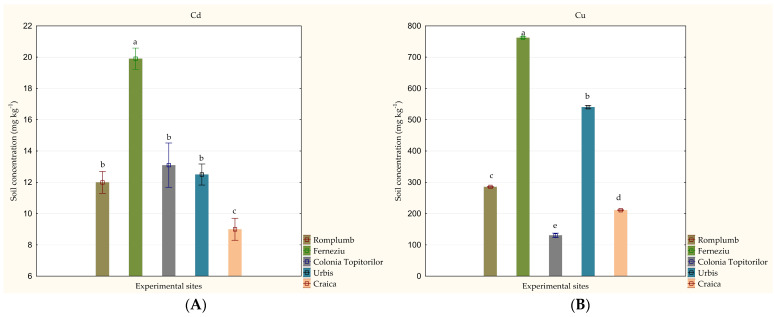

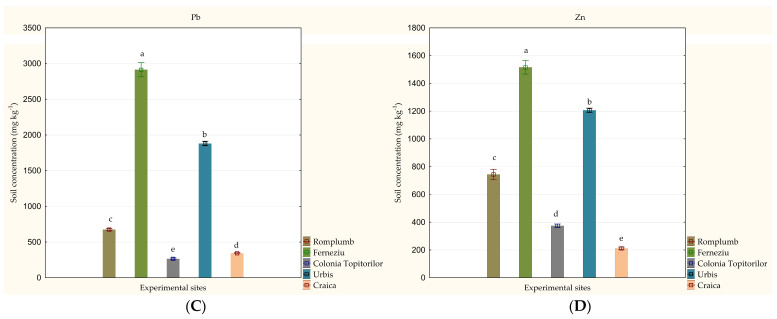
Variation in soil heavy metal concentration among the studied experimental sites (mg kg^−1^): (**A**) Cd, (**B**) Cu, (**C**) Pb and (**D**) Zn. Concentrations are given as an average of five replications ± standard deviation. Effects were accepted as statistically significant if *p* ≤ 0.05. Values marked with a common letter are not significantly different, according to the Tukey HSD Test. (Source: Original).

Soil analysis showed high variations in HM content among the five experimental sites studied, ranging between: 9.0 and 19.9 mg kg^−1^ for Cd, 130.6 and 762.3 mg kg^−1^ for Cu, 272.3 and 2913.3 mg kg^−1^ for Pb; 211.7 and 1515.6 mg kg^−1^ for Zn (Figure 3). The highest values for all of the four HMs studied were detected in Ferneziu (*p* < 0.001). The soil from Craica showed the lowest content for Cd (*p* < 0.001) and Zn (*p* < 0.001), while the lowest values for Cu and Pb were reached in Colonia Topitorilor (*p* < 0.001).

### 3.2. HMs Contents in Plant Tissues

Plant analysis showed high variations in HM content among the five experimental sites studied, and between RJ plant tissues (Table 3). Generally, the highest content in Cd and Cu were reached in Ferneziu, Craica and Urbis, while for Pb and Zn, the highest values were recorded for Romplumb (except for Pb content in leaf). Considering the HM content found in plant tissues, our results show that the highest Cd content was recorded in roots and stems from Ferneziu, a result closely followed by that found in Craica (*p* > 0.05) and significantly higher than that found in the roots and stems from the other three sites. The highest concentrations of Cd in leaves were recorded in Craica, a result significantly higher than that found in the other four sites (*p* < 0.001). Higher values were also reached in Craica for the Cu content found in the roots and stems of RJ, which were very significant from a statistical point of view. As shown in Table 3, our results highlighted that the highest concentrations of Pb among all plant tissues analyzed were recorded in Romplumb, where the values reached were significantly higher than those recorded for the other four experimental sites. Higher values were also reached in Romplumb for the Pb content of roots and stems, while the highest content of Pb found in the leaves of this species was reached in Craica. 

### 3.3. HM Dynamics in Reynoutria japonica Houtt.-Soil Interactions

#### Translocation Factor Assessment

The values achieved for the translocation factor (TF) are presented in Table 4. Our results show values higher than one for Cd, with the maximum mean obtained for Urbis (*p* > 0.05). The values recorded for the other three HMs were lower than one, showing the following trend:

▪ for Cu: Urbis > Colonia Topitorilor > Romplumb > Craica > Ferneziu;
▪ for Pb: Romplumb > Urbis = Craica > Ferneziu > Colonia Topitorilor;
▪ for Zn: Craica > Urbis > Romplumb > Ferneziu > Colonia Topitorilor.

The values achieved for the bioconcentration factor (BCF) are presented in Table 5. Variations in BCF values are recorded among the five experimental sites, but this indicator generally follows the same decreasing trend as follows: Cd > Zn > Cu > Pb. The BCF value for Cd, calculated for the stems and leaves of RJ, was higher than one on all of the five experimental sites, while for the roots, the only site which scored a BCF value higher than one was Craica (*p* < 0.001). High values for the BCF were also recorded for the Zn found in the roots from Craica, Romplumb and Colonia Topitorilor, and also for the Zn found in the stems and leaves from Craica. 

## 4. Discussion

*Reynoutria japonica* Houtt. is an urbanophile plant species, originally from Asia, very aggressive and especially spread to roadways, railways and waterways, where it usually forms compact, thick layers [21]. RJ was also reported to colonize polluted environments, including those with HM [32,33]. Therefore studies aiming to investigate this species’ strategies to cope with HM-polluted soils are of particular interest.

Generally, the on-field studies aiming to evaluate HM transfer into RJ reported soil HM concentrations up to: 6.75 mg kg^−1^ for Cd [33,34], 89.7 mg kg^−1^ for Cu [32,33], 178 mg kg^−1^ for Pb [34,35] and 581 mg kg^−1^ for Zn [32,33,34], respectively. The concentrations of HM found in the soil samples collected from the five sites considered in this research were higher than those reported by these studies, in the range 9–19.9 mg kg^−1^ for Cd, 130.6–762.3 mg kg^−1^ for Cu, 272.3–2913.3 mg kg^−1^ for Pb, and 211.7–1515.6 mg kg^−1^ for Zn (Figure 3). The mean values of HM in soil samples collected from the study sites exceeded the threshold limit values for intervention established by the Romanian legislation (5 mg kg^−1^ for Cd; higher than 20 mg kg^−1^ for Cu; 250 mg kg^−1^ for Pb; higher than 100 mg kg^−1^ for Zn), demonstrating a high degree of HM contamination found in the investigated sites. Still, the results reached in this study through the vegetation surveys showed that RJ occupied a high share in the vegetation cover of the five sites from Baia Mare, Romania (between 20 and 80%; Table 1), highlighting that this species is able to tolerate higher concentrations of HM than those reported by previous studies. Interestingly, RJ showed the highest percentage of vegetation cover in the Ferneziu and Urbis sites; these sites recorded the highest concentration of HM in the soils of all the sites considered in this study. These variations in soil HM content could be partially explained by the location of these two sites between the two former industrial companies Romplumb Baia Mare and Cuprom Baia Mare (Figure 1; Table 1), while the other three sites were located at the edge of just one of the two major sources of contamination, or at a greater distance. In contrast, the lowest share of RJ species in the vegetation cover was reached in the site with the lowest level of HM contamination, namely Craica. These results could be explained by RJ’s ability to grow on highly anthropized soils, where other native plant species are usually unable to grow or adapt well. As such, RJ could have become the dominant species in the investigated sites thanks to the competitive exclusion pattern. 

The chemical properties of the soils analyzed among the five sites included in our research revealed significantly different characteristics (Table 2 and Table 3). The soil pH in the experimental sites from Baia Mare ranged from very strong acid (Craica), moderate acid (Romplumb, Colonia Topitorilor) and neutral (Urbis). Other researchers have also pointed out the wide pH ranges in habitats colonized by RJ and HM-polluted soils. Palmer [36] reported a pH range between 3 and 8, while Vanderhoeven et al. [37] mentioned values between 4.4 and 7.3. Such a wide range in pH could be explained by the character of the soil substrate and the concentration of soluble alkaline mineral elements [33]. Variations were detected also considering the organic C, which ranged between very high content (Ferneziu and Urbis; Table 3), moderate (Craica) and very low (Romplumb and Colonia Topitorilor). In general, these sites also showed a relatively low content of total N. The maximum concentration of P was found in Urbis, a value very high compared to that recorded for the other four experimental sites, 552 ppm in Urbis compared to 1 ppm in Craica or 2 ppm in Romplumb (*p* < 0.001). 

It is well known that excessive soil phosphorus combined with a pH > 6.5 reduces the plant’s ability to take up required micronutrients (particularly Fe and Zn). These kinds of soil conditions were found in Urbis and could explain the high share of RJ in this location. Dassonville et al. [38] pointed out that the rhizomes of RJ may grow deep into the natural mineral horizons, where they reach the alkaline elements, and this could be the mechanism which allowed RJ to grow and develop in the soil conditions found in Urbis. Furthermore, the high share of RJ in the vegetation cover from Ferneziu and Urbis could have influenced soil organic C, which recorded significantly higher values in these two locations. Some researchers have already pointed out that RJ could influence soil properties by enhancing soil organic C and nutrient concentrations (Ca, Cu, N, P and Zn) [39,40]. However, little is known about these interactions so far. For example, Bullock and Gregory [41] reported contradictory results, concluding that organic C can be low in many urban soils. Still, given the high biomass produced by RJ in the two sites analyzed in this study (Table 1 and Figure 2), and considering the greater C, Ca concentrations and C/N, C/P ratios compared to resident vegetation reported by previous studies [39,42,43], we could partially explain these variations in the topsoil physicochemical properties of the five sites. On the other hand, one aspect that should be considered is the nature of these soils. As previously mentioned in Table 1, the five sites considered in this study are classified as brownfields, with anthropic/urban soil, which also contributes to these variations in topsoil physicochemical properties. 

Barberis et al. [44] previously demonstrated the high tolerance of RJ to metal stress, pointing out that plant response to HM pollution depends on HM identity. Plant analysis (as previously presented in Table 3) showed that the highest contents of Cd and Cu were recorded in Craica. Although Cu is an essential element for photosynthesis and other processes [45], the values found in RJs tissues are considered to be toxic, since they exceed the maximum permissible level for Cu concentration in plant species (10 mg kg^−1^ ). These results are interesting, since Craica was the least contaminated site. One explanation could be provided by the soil’s physicochemical properties, since previous studies have demonstrated that the chemical forms and bioavailability of HM are influenced by some environmental parameters, such as soil pH, cation exchange capacity and organic C [46,47]. For example, a lower soil pH could reduce HM adsorption and increase mobility [48], while high levels of organic C could increase the solubility and availability of HM [49]. In the particular case of Craica and Romplumb sites, the higher contents in Cu, Cd and even Pb found in RJ tissues could be explained by the soil pH since previous studies evidenced that low pH soils favored the bioavailability of these trace elements [50]. 

The results reached in this study showed that generally, the highest contents of the four HMs studied were found in the plant’s roots (Cu, Pb and Zn), except for Cd, which showed higher values in the leaves. Other researchers also pointed out similar compartmentalization of HM among RJs tissue [51,52]. A different compartmentalization was observed for Pb, such that the highest values of this trace metal were detected in the root, followed by the stem, and then the leaves. According to Mitrić et al. [53], plants with phytoremediation ability are characterized by the accumulation of Pb in the root system and lower values to the above-ground parts (stems, leaves, and flowers). The content in Pb found in RJs roots varied between 103 mg kg^−1^ in Urbis and 208 mg kg^−1^ in Romplumb. These values (only concerning the Pb in root) are even 20 times higher than the natural concentrations of Pb in plants (which ranges between 5 and 10 mg kg^−1^ according to Radojević and Bashin [54]). 

Thus, we can confirm through our results that this species is not only able to adapt to HM-polluted soils but can also extract substantial amounts of HM from soils. 

**Figure 4:**
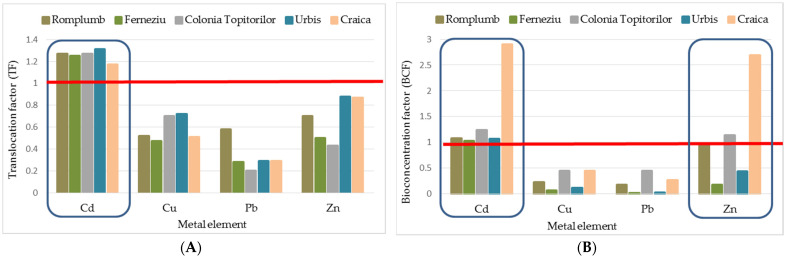
Correlation between the type of HM, experimental site and TF (**A**), and BCF (**B**). The red line marks the reference value (equal to 1) for the interpretation of TF and BCF.

In order to understand and define HM dynamics in RJ–soil interactions, we studied this species’ ability to translocate and bioaccumulate the metals from roots through shoots and leaves further. This investigation was performed by calculating the translocation factor (TF) and the bioconcentration factor (BCF). The values achieved for these indicators show an enhanced ability of RJ to bioaccumulate heavy metals (Table 4 and Table 5; Figure 4A,B). According to Takarina et al. [55], a value less than one shows that a plant species can be used as a phytostabilizer (meaning that a metal concentration is found in the underground part of the plant), while on values higher, the species could be used as phytoextractors (plants able to transport and concentrate metals from the soil to the above-ground parts of plants).

In the case of Pb, all of the TF and BCF values were below one (between 0.2 and 0.58 for TF; and 0.01 and 0.44 for BCF), with the highest values found in the root, indicating that RJ could have a role as a phytostabilizer for soils polluted with this trace metal. The highest values (>1) for both indicators were found for Cd on all of the five experimental sites studied (Figure 4), showing a strong translocation potential for this trace element from soil–root–above-ground plant parts. 

TF values below one were also recorded for Cu and Zn. Still, all of the BCF values calculated for Zn (average for the three plant tissue analyzed) were higher than one in Craica and Colonia Topitorilor and for the root from Romplumb. These results show that RJ is able to transport and concentrate Zn from the soil to the above-ground parts of plants, revealing this species’ suitability as phytoextractors (for Cd and Zn). Similar responses were also reported by Ibrahimpašić et al. [51].

## 5. Conclusions

This study has demonstrated that RJ, besides its aggressive colonization of different environments, thus posing enormous threats to native biodiversity, can tolerate, extract and translocate high amounts of HM among plant tissues. The values obtained for the TF and BCF indicate an efficient movement of Cd and Zn to the above-ground parts of the plant, while Pb was the least bioaccumulated trace element. Furthermore, it seems that RJ’s response to the varying range of HM contamination found at the five sites was dependent on HM identity: Cd was found in high amounts in the above-ground part of the plant (stem and leaves), while Cu, Pb and Zn reached the highest values (with a few exceptions) in the root. The results reached in this study suggest that the bioavailability of Cd, Cu, Pb and Zn could have been influenced by soil physicochemical properties (particularly the pH), which in turn, was shown to be influenced by the proportion of RJ in the vegetation cover. A positive correlation was observed between RJ and organic C, which recorded the highest values in Ferneziu and Urbis (where RJ covered 65% and 80%, respectively). The present study not only revealed RJ’s ability to cope with the high concentrations of HM found in the soils but also to grow and produce high biomass under extreme soil conditions (excessive P) where most of the native species were unable to grow. These results are of particular concern for restoration programs. Given the complexity of these interactions, we recommend further investigation to understand the entire spectra of dynamics occurring between RJ–soils–HM at contaminated sites better.

## Figures and Tables

**Table 1:** Characterization of the experimental sites.

No.	Experimental Site	Type of Habitat	Soil Type	RJ Coverage
1	Romplumb	Brownfield, riparian ecosystem	Anthropic protosoil (anthropic/urban soil)	50%
2	Ferneziu	Brownfield, riparian ecosystem	Anthropic protosoil (anthropic/urban soil)	65%
3	Colonia Topitorilor	Brownfield, riparian ecosystem	Anthropic protosoil (anthropic/urban soil)	40%
4	Urbis	Brownfield, riparian ecosystem	Anthropic protosoil (anthropic/urban soil)	80%
5	Craica	Brownfield, riparian ecosystem	Anthropic protosoil (anthropic/urban soil)	20%

**Table 2:** Soil chemical composition.

Indicator	Experimental Site				
	Romplumb	Ferneziu	Colonia Topitorilor	Urbis	Craica
pH (H_2_O)	5.26 ± 0.01 ^cd^	6.31 ± 0.01 ^b^	5.52 ± 0.01 ^c^	7.31 ± 0.01 ^a^	4.84 ± 0.01 ^d^
Organic C (%)	0.67 ± 0.007 ^e^	6.86 ± 0.3 ^a^	0.88 ± 0.005 ^d^	3.42 ± 0.1 ^b^	2.12 ± 0.005 ^c^
N (%)	0.057 ± 0.005 ^c^	0.621 ± 0.00 ^a^	0.054 ± 0.002 ^c^	0.218 ± 0.02 ^b^	0.204 ± 0.01 ^b^
Mobile P (ppm)	2 ± 0.01 ^c^	70 ± 2.31 ^b^	4 ± 1.01 ^c^	552 ± 9.2 ^a^	1 ± 0.001 ^c^
Clay (0–2 μm; %)	32.52 ± 3.16 ^a^	17.65 ± 3.80 ^b^	33.85 ± 6.35 ^a^	24.95 ± 5.15 ^a^	35.95 ± 7.25 ^a^
Fine sand (50–200 μm; %)	29.91 ± 8.52 ^a^	32.54 ± 4.65 ^a^	29.93 ± 3.81 ^a^	36.24 ± 6.26 ^a^	24.95 ± 6.06 ^a^
Coarse sand (200–2000 μm; %)	15.29 ± 4.81 ^b^	34.66 ± 5.76 ^a^	11.97 ± 2.07 ^bc^	19.89 ± 2.60 ^b^	5.1 ± 1.1 ^c^

Concentrations are given as an average of five replications ± standard deviations. Organic C—soil organic carbon. N—Total nitrogen content. Mobile P—mobile phosphorous. Effects were accepted as statistically significant if *p* ≤ 0.05. Values within the same row followed by a common letter are not significantly different, according to the Tukey HSD Test.

**Table 3:** Variation in heavy metal content (Cd, Cu, Pb and Zn; mg kg^−1^) in *Reynoutria japonica* Houtt. plant tissues among the studied experimental sites.

Indicator	Experimental Site				
	Romplumb	Ferneziu	Colonia Topitorilor	Urbis	Craica
Cd (root)	10.5 ± 1.30 ^bc^	17.9 ± 4.52 ^a^	11.1 ± 1.71 ^bc^	11.3 ± 3.94 ^b^	17.3 ± 5.68 ^a^
Cd (stem)	13.3 ± 1.52 ^b^	22.0 ± 5.29 ^a^	14.0 ± 2.00 ^b^	14.3 ± 4.61 ^b^	20.28 ± 6.65 ^a^
Cd (leaf)	15.3 ± 2.08 ^c^	22.0 ± 4.58 ^b^	24.0 ± 7.21 ^b^	14.0 ± 3.60 ^c^	40.9 ± 13.41 ^a^
Cu (root)	98.4 ± 7.16 ^b^	90.6 ± 2.06 ^c^	59.1 ± 3.86 ^e^	70.4 ± 1.92 ^d^	140.6 ± 3.05 ^a^
Cu (stem)	51.3 ± 1.52 ^b^	43.0 ± 1.00 ^c^	42.0 ± 2.00 ^c^	51.66 ± 2.88 ^b^	71.7 ± 1.55 ^a^
Cu (leaf)	55.0 ± 4.00 ^c^	50.6 ± 1.15 ^c^	81.0 ± 5.29 ^b^	92.3 ± 2.51 ^a^	78.58 ± 1.70 ^b^
Pb (root)	208.9 ± 3.37 ^a^	106.2 ± 2.27 ^d^	121.1 ± 10.44 ^c^	103.2 ± 11.62 ^d^	156.6 ± 7.63 ^b^
Pb (stem)	122.6 ± 11.01 ^a^	30.3 ± 1.52 ^c^	25.3 ± 3.21 ^c^	30.33 ± 3.51 ^c^	45.6 ± 2.51 ^b^
Pb (leaf)	42.6 ± 1.52 ^c^	20.6 ± 0.57 ^d^	14.3 ± 0.57 ^e^	57.0 ± 6.42 ^b^	86.0 ± 4.21 ^a^
Zn (root)	828.0 ± 8.18 ^a^	399.7 ± 13.90 ^e^	658.0 ± 6.55 ^b^	535.3 ± 9.86 ^d^	568.6 ± 6.86 ^c^
Zn (stem)	587.2 ± 5.80 ^a^	204.6 ± 2.18 ^e^	284.8 ± 2.83 ^d^	473.7 ± 8.73 ^c^	498.3 ± 5.97 ^b^
Zn (leaf)	700.3 ± 13.57 ^a^	223.3 ± 7.76 ^e^	352.6 ± 3.78 ^d^	626.3 ± 14.15 ^c^	642.5 ± 7.69 ^b^

Concentrations are given as an average of five replications ± standard deviations. Cd (root, stem, and leaf)—cadmium in root, stem, and leaf. Cu (root, stem, and leaf)—copper in root, stem, and leaf. Pb (root, stem, and leaf)—lead in root, stem and leaf. Zn (root, stem, and leaf)—zinc in root, stem, and leaf. Effects were accepted as statistically significant if *p* ≤ 0.05. Values within the same row followed by a common letter are not significantly different, according to the Tukey HSD Test.

**Table 4:** The potential of RJ in accumulating HM (Cd, Cu, Pb and Zn) according to the TF.

Indicator	Experimental Site				
	Romplumb	Ferneziu	Colonia Topitorilor	Urbis	Craica
Cd	1.27 ± 0.18 ^a^	1.25 ± 0.18 ^a^	1.27 ± 0.17 ^a^	1.31 ± 0.24 ^a^	1.17 ± 0.01 ^a^
Cu	0.52 ± 0.05 ^b^	0.47 ± 0.01 ^bc^	0.70 ± 0.03 ^a^	0.72 ± 0.01 ^a^	0.51 ± 0.02 ^bc^
Pb	0.58 ± 0.06 ^a^	0.28 ± 0.01 ^bc^	0.20 ± 0.01 ^c^	0.29 ± 0.03 ^b^	0.29 ± 0.06 ^b^
Zn	0.70 ± 0.04 ^c^	0.50 ± 0.02 ^d^	0.43 ± 0.02 ^d^	0.88 ± 0.03 ^a^	0.87 ± 0.03 ^a^

Effects were accepted as statistically significant if *p* ≤ 0.05. Values within the same row followed by a common letter are not significantly different, according to the Tukey HSD Test.

**Table 5:** The potential of RJ in accumulating HM (Cd, Cu, Pb and Zn) according to the BCF.

Indicator	Experimental Site				
	Romplumb	Ferneziu	Colonia Topitorilor	Urbis	Craica
Cd (root)	0.87 ± 1.00 ^a^	0.89 ± 0.22 ^a^	0.84 ± 0.13 ^a^	0.94 ± 0.35 ^a^	1.92 ± 0.68 ^b^
Cd (stem)	1.11 ± 0.12 ^b^	1.10 ± 0.13 ^b^	1.06 ± 0.07 ^b^	1.14 ± 0.23 ^b^	2.25 ± 0.73 ^a^
Cd (leaf)	1.27 ± 0.17 ^b^	1.10 ± 0.22 ^b^	1.83 ± 0.55 ^b^	1.12 ± 0.28 ^b^	4.54 ± 1.48 ^a^
Cu (root)	0.33 ± 0.02 ^c^	0.11± 0.01 ^d^	0.44 ± 0.03 ^b^	0.12 ± 0.00 ^d^	0.66 ± 0.01 ^a^
Cu (stem)	0.17 ± 0.00 ^b^	0.05 ± 0.03 ^c^	0.31 ± 0.01 ^a^	0.09 ± 0.02 ^c^	0.33 ± 0.01 ^a^
Cu (leaf)	0.18 ± 0.01 ^c^	0.06 ± 0.02 ^d^	0.61 ± 0.04 ^a^	0.16 ± 0.00 ^c^	0.37 ± 0.01 ^b^
Pb (root)	0.30 ± 0.01 ^b^	0.03 ±0.01 ^c^	0.44 ± 0.03 ^a^	0.05 ± 0.01 ^c^	0.44 ± 0.05 ^a^
Pb (stem)	0.17 ± 0.01 ^b^	0.03 ±0.02 ^c^	0.08 ±0.01 ^b^	0.01 ± 0.00 ^bc^	0.12 ± 0.00 ^a^
Pb (leaf)	0.06 ± 0.02 ^c^	0.01 ±0.00 ^d^	0.06 ±0.02 ^e^	0.02 ± 0.00 ^b^	0.24 ± 0.05 ^a^
Zn (root)	1.11 ± 0.018 ^c^	0.26 ± 0.01 ^e^	1.74 ± 0.05 ^b^	0.43 ± 0.02 ^d^	2.68 ± 0.06 ^a^
Zn (stem)	0.78 ± 0.08 ^b^	0.13 ± 0.01 ^d^	0.75 ± 0.05 ^b^	0.38 ± 0.03 ^c^	2.35 ± 5.97 ^a^
Zn (leaf)	0.93 ± 0.02 ^b^	0.14 ± 0.00 ^d^	0.93 ± 0.01 ^b^	0.51 ± 0.01 ^c^	3.03 ± 0.30 ^a^

Effects were accepted as statistically significant if *p* ≤ 0.05 (HS, confidence 99.9%). Values within the same row followed by a common letter are not significantly different, according to the Tukey HSD Test.

## Data Availability

Not applicable.
